# Effect of Micro and Nano Boron Nitride on Thermal Conductivity and Electrical Properties of Mica Tape

**DOI:** 10.3390/ma19091821

**Published:** 2026-04-29

**Authors:** Yu Feng, Minhao Tian, Xuesong Chen, Wenchao Zhang, Sergey A. Maksimenko, Dong Yue, Yuanhang Yao

**Affiliations:** 1 Key Laboratory of Engineering Dielectrics and Its Application, Ministry of Education, Harbin University of Science and Technology, Harbin 150080, China; 2 School of Electrical and Electronic Engineering, Harbin University of Science and Technology, Harbin 150080, China; 3 Institute for Nuclear Problems, Belarusian State University, 220006 Minsk, Belarus

**Keywords:** mica tape, boron nitride, thermal conductivity, dielectric properties, thermal model

## Abstract

**Highlights:**

**What are the main findings?**

- Thermal conductivity and dielectric properties of h-BN-modified mica tape with different particle sizes.
- A two-dimensional heat transfer model was constructed with particle size as the core variable.

**What are the implications of the main findings?**

- Investigating the influence of h-BN particle size on the thermal conductivity of mica tape.
- The thermal conductivity model aids in predicting the thermal conductivity coefficient of mica tape for subsequent applications.

**Abstract:**

As the power industry continues to advance rapidly, large-scale generators are evolving toward higher voltage levels and greater capacity. The heat accumulation associated with high voltage and large capacity accelerates the aging of the main insulation. It is necessary to enhance the thermal conductivity (*λ*) and dielectric properties of existing main insulation materials. This work focuses on investigating the effects of varying addition levels of two different-sized BN particles on the *λ* and dielectric properties of the mica tape composite dielectric. The experimental findings demonstrate a progressive enhancement in the *λ* of the mica tape corresponding to the incremental addition of h-BN concentration. When the doping concentration reaches 20 wt.%, the *λ* of the two h-BN-doped mica tape (h-BN/MT) reaches a maximum of 0.382 W/(m·K), 0.4 W/(m·K), respectively, which enhances the *λ* of the contrasting pure mica tape (0.199 W/(m·K)) by 91.95% and 101.01%, respectively. In terms of electrical insulation properties, both sizes of h-BN/MT perform well, with breakdown strength above 32 kV/mm. Furthermore, the second-order thermal conductivity model of mica tape doped with different sizes of h-BN was constructed by combining the Halpin–Tsai model with the Series model, which allows the calculation of *λ* of mica tape composites doped with different sizes of h-BN. This work provides a novel structural design approach for preparing mica tape composite dielectric that simultaneously exhibits high *λ* and high insulation properties.

## 1. Introduction

In the context of the swift advancement of the worldwide power industry, generator technology is constantly developing towards high voltage, miniaturization and large capacity [1,2,3,4]. As a key insulating material for generators, mica tape is mainly used to wrap stator windings to form an insulating protective layer. During the operation of the generator, the stator windings will generate a large amount of heat due to the continuous energization. However, the traditional mica tape is difficult to conduct this heat effectively due to its poor *λ*, which not only affects the operational stability of the equipment, but also shortens the service life of the generator. Therefore, the development of mica tape with high *λ* is of great significance for the stable operation of the equipment and the extension of its service life [5].

Mica tape consists of a three-layer structure of fiberglass cloth, epoxy resin (EP) adhesive and mica paper, and EP has the lowest *λ* of the three. There are two main means to improve the *λ* of EP [6,7,8,9,10,11,12]: the first is intrinsically modified EP. Wang et al. [13] introduced rigid groups of 4,4′-dihydroxybiphenyl into the backbone of EP by ring-opening polymerization (ROP) to change the molecular structure of EP. The *λ* of the cured EP reaches 0.33 W/(m·K); its *λ* is 165% of the *λ* of conventional EP. Wang et al. [14] prepared naphthalene liquid crystal epoxy resin (LCER) with high intrinsic *λ* of 0.40 W/(m·K), which is about 2.2 times higher than that of conventional EP. Zhang et al. [15] synthesized liquid crystal epoxy resins containing biphenyl liquid crystal units via an efficient one-step reverse synthesis method, and cured liquid crystal epoxy monomers containing biphenyl liquid crystal units with commercial bisphenol A-type epoxy resins. The *λ* of the biphenyl mesocrystalline units cured with BPAF reached 0.38 W/(m·K), which was nearly double that of the DDM-cured commercial bisphenol A-type EP (0.20 W/(m·K)). Zhang et al. [16] synthesized a linear fluorinated epoxy copolymer (poly(PFS-co-GMA)) and used it to prepare a liquid crystal epoxy resin with a *λ* of 0.40 W/(m·K), which is a 200% increase in *λ*. The second type is directly doped EP. Jia et al. [17] prepared bismuth oxybromide (BiOBr) fillers by hydrothermal synthesis and composited them with EP. The *λ* of BiOBr/EP is highest at 1 wt.% filler (0.22 W/(m·K)), which exhibits a nearly 30% enhancement compared to unmodified EP. Akhtar et al. [18] treated the surface of alumina particles with 3-aminopropyltriethoxysilane (A-Al_2_O_3_); graphene was surface-modified with 3-glycidylpropyltrimethoxysilane (GPTMS) to produce G-graphene. When the volume fraction of the filler reaches 50%, the *λ* of the composite is enhanced by more than 8 times over unmodified EP. Tian et al. [19] used a composite material prepared by mixing 45 μm and 3 μm alumina composite fillers in a 3:2 ratio, achieving a thermal conductivity improvement of approximately 10% to 40%. The *λ* of the novel material reaches 0.27 W/(m·K), which exhibits a 157% enhancement factor over EP (0.17 W/(m·K)). The preparation process of intrinsic EP is complicated and the output is low, which is only suitable for laboratory research and cannot be applied in engineering at this stage, while the preparation process of direct doped EP is simple and more common in engineering applications nowadays.

Currently, common thermally conductive fillers include: alumina [20,21,22,23,24], magnesium oxide [25,26,27], silicon carbide [28,29,30], aluminum nitride [31,32,33], and boron nitride [34,35,36,37,38]. Among them, boron nitride is widely used in the thermal conductivity modification of EP due to its high *λ* and excellent dielectric insulation properties. Nowadays, most of the researchers consider the effect of the concentration and dimension of boron nitride on the *λ* and insulating properties of EP [39,40], neglecting the effect of its own size on EP. As the continuous phase inside the mica tape, the EP needs to be closely combined with the fiberglass cloth, and the size of the boron nitride grains inside the EP needs to match with the pore size of the fiberglass cloth in order to be conductive to the composition of the thermally conductive pathway in the tape. Therefore, it is very necessary to investigate the size of boron nitride on the *λ* as well as the insulating properties of mica tape.

In existing research, the size of mica tape thermal interface materials has been largely overlooked. This study investigates the effect of particle size in mica tape fillers as a variable—an area previously unexplored by scholars. This study represents a pioneering investigation into the dimensional characteristics of thermal conductive fillers within mica tapes. We selected two composite materials from five types of mica tape composite insulating materials doped with h-BN. These two materials exhibit a relatively pronounced performance gap, facilitating comparison. Mica tape composites with high *λ* and good dielectric insulation properties were prepared by combining fiberglass cloth, high thermal conductivity h-BN/EP, and mica paper through physical blending and hot pressing techniques. The microstructure of mica tape composites was investigated, and their *λ*, breakdown field strength, electrical conductivity, relative permittivity, and dielectric loss were analyzed. Due to the complex structure of mica tape, conventional thermal conductivity models cannot accurately predict its behavior. Therefore, constructing a two-dimensional thermal conductivity model is particularly crucial. Additionally, considering the impact of particle size variations in thermal conductive fillers on thermal conductivity, this study opts to refine the Halpin–Tsai model to address gaps in existing research. The HT-S model’s advantages over existing models primarily lie in: (1) Its ability to handle the complex structure of mica tape. Existing thermal conductivity models can only intelligently analyze individual sections, failing to accurately predict the overall behavior. (2) The incorporation of particle size as a critical variable. Existing thermal conductivity models do not adapt when the size of thermal fillers is changed, meaning thermal conductivity predictions cannot adjust accordingly. This results in inaccurate predictions. This provides a reference basis for subsequent studies on the λ of doped mica tape and the selection of filler sizes for high-thermal-conductivity composite materials.

## 2. Materials and Methods

### 2.1. Materials

The adhesive selected is WSR618 (E51) epoxy resin produced by Nantong Xingchen Synthetic Materials Co., Ltd. (Nantong, China). The curing agent selected is methyl hexahydrophthalic anhydride (MHHPA) provided by Changzhou Runxiang Chemical Co., Ltd. (Changzhou, China). The accelerator selected is 2,4,6-tri(dimethylamino)phenol (DMP-30) from Kunshan JiuLiMei Electronic Materials Co., Ltd. (Kunshan, China). The thermal conductive filler selected is h-BN from Beijing Dekedao Jin Technology Co., Ltd. (Beijing, China), with sizes of 50 nm, 500 nm, 1–3 μm, 5 μm, and 10 μm. Mica paper sheets (DMP-M160) are sourced from Pingjiang County Weipai Mica Insulation Materials Co., Ltd. (Pingjiang, China). Fiberglass cloth is sourced from Rudong Tiancheng Glass Fiber Co., Ltd. (Nantong, China).

### 2.2. Preparation of h-BN/Epoxy Resin Composites

**Figure 1:**
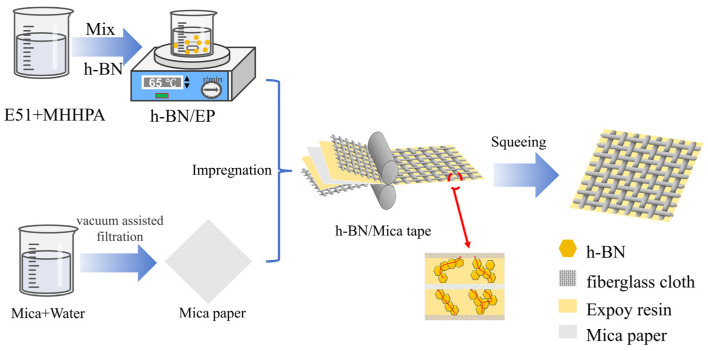
h-BN/MT and h-BN/EP preparation diagram.

First, place E51 in an oven at 65 °C for half an hour to ensure good flowability. Then, weigh an appropriate amount of E51 into a beaker and add MHHPA to the beaker in a specific ratio. Subsequently, add the weighed h-BN at 65 °C and stir for 6 h at an appropriate speed (adjusted according to the mass of the solid–liquid mixture in the beaker). Five composites with different mass fractions were prepared by controlling the mass of h-BN (Figure 1).

### 2.3. High Thermal Conductivity Mica Tape Preparation Process

Mica tape consists of three parts: fiberglass cloth, adhesive, and mica paper. Therefore, mica paper must be prepared before mica tape can be manufactured. Mica paper is primarily composed of overlapping mica flakes. First, an appropriate amount of mica is added to 500 mL of distilled water, and mechanical stirring and ultrasonic agitation are used to uniformly disperse the mica flakes in the distilled water. Subsequently, vacuum-assisted filtration and hot pressing technology are employed to layer the mica flakes and form mica paper. Place the mica paper between two layers of h-BN/EP composite filler-filled fiberglass cloth. Use a flat plate vulcanizer to compress the mica tape at 65 °C and 3 MPa pressure for 20 min. Finally, remove it and place it in an oven for curing, thereby obtaining a smooth-surfaced h-BN/MT composite material. Excess EP is squeezed out of the mica tape by hot pressing to ensure the thickness and density of the tape (Figure 1).

### 2.4. Testing Methods

After applying a gold coating to all mica tape samples, a scanning electron microscope (SEM) was used to observe the microstructural features of the cross-sections of the mica tape samples. Mica tape samples were tested for *λ* using a DRL-III thermal conductivity tester with the heat flux method. Prior to testing, PT-M25 composite thermal grease was uniformly applied to both sides of the mica tape. The *E_b_* of the samples was measured according to the ASTM-D149 standard [41]. The conductivity (*γ*) of the mica tape was measured using a laboratory-built three-electrode conductivity testing system. The dielectric constant (*ε_r_*) and dielectric loss (tan*δ*) of the mica tape samples were measured using a wideband dielectric spectrometer analyzer at 25 °C within the frequency range of 1–10^6^ Hz.

## 3. Results and Discussion

### 3.1. Microstructure of h-BN/MT

**Figure 2:**
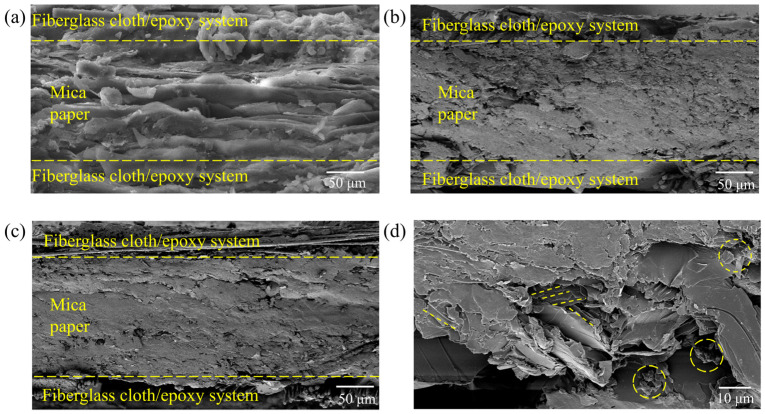
Electron microscopy of mica tape cross-section: (**a**) unmodified mica tape, (**b**) modified mica tape, (**c**) modified mica tape, (**d**) localized enlarged view.

The cross-sectional images of h-BN/MT were observed using a scanning electron microscope (SEM) as shown in Figure 2. As shown in Figure 2a, the mica tape impregnated with unmodified epoxy resin exhibits excellent impregnation results. In contrast, the mica tape impregnated with modified epoxy resin shows insufficient impregnation, yet the overall impregnation effect remains satisfactory as demonstrated in Figure 2b,c. By expanding the localization range, it was discovered that hexagonal boron nitride forms interconnected structures within the mica ribbon, thereby establishing continuous thermal conduction pathways Figure 2d. This lays a solid foundation for enhancing *λ* in mica ribbon composites.

### 3.2. Thermal Conductivity of h-BN/MT Composites

**Figure 3:**
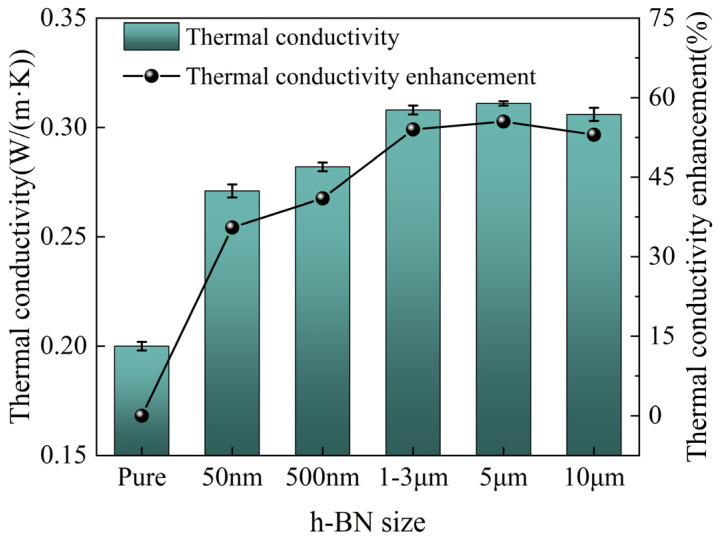
The *λ* of five different h-BN/mica and their enhancement.

To investigate the effect of different h-BN sizes on the *λ* value of mica tape, preliminary experiments were first conducted to optimize the h-BN size. At the same mass fraction (10 wt.%), five different sizes of h-BN were selected to investigate the *λ* of mica tape, as shown in Figure 3. Among these, the 5 μm grain-filled mica tape exhibits the highest *λ*, reaching 0.31 W/(m·K). Compared to the unmodified mica tape, its *λ* has increased by 55.5%. As the particle size of h-BN increases, the *λ* of the mica tape first increases and then remains constant. This is primarily because, at a fixed filling content, larger h-BN particles facilitate overlapping to form thermal conduction pathways. Since all three types of large-size h-BN can fully overlap to form thermal conduction pathways, the *λ* of the mica tape remains essentially unchanged due to the fixed filling volume of h-BN.

Finally, two sizes of h-BN were selected from the five sizes, namely 50 nm and 10 μm. 50 nm and 10 μm h-BN were selected because the difference in size between the two was large, and the influence of size on the *λ* of the doped composites could be more intuitively demonstrated. In addition, although the 5 μm size doped h-BN/MT has the highest *λ*, the difference with the 10 μm size doped *λ* is not large, for the purpose of ensuring that the size’s impact on *λ* can be more fully demonstrated, so 10 μm was selected.

**Figure 4:**
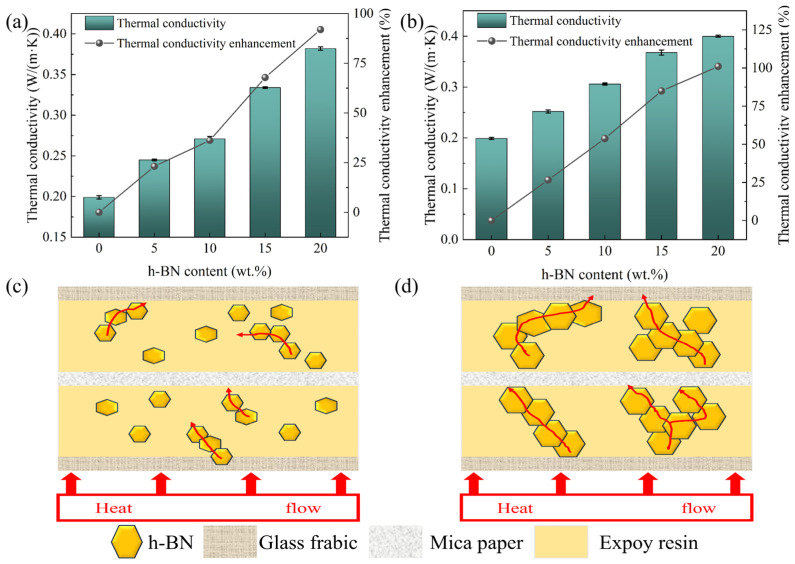
The *λ* and thermal conductivity enhancement rate of h-BN/MT with different sizes: (**a**) 50 nm, (**b**) 10 μm. Schematic diagram of two-dimensional heat transfer mechanism (**c**) small-size h-BN, (**d**) large-size h-BN.

After preexperimental investigation, two sizes (50 nm, 10 μm) h-BN/MT with different mass fractions were prepared, and their thermal conductivities are shown in Figure 4. As shown in Figure 4, the *λ* of both size h-BN/MT composites exhibited an enhancement with rising h-BN filling amount. Among them, the 10 μm size h-BN/MT has the highest *λ* (0.4 W/(m·K)) at a filler level of 20 wt.%, representing a 101.01% enhancement compared to unmodified mica tape. The underlying rationale pertains to considering that the h-BN exhibits excellent dispersion in EP and lapped with each other to form a good thermal conductivity network, which increases the *λ* of the EP and thus enhances the *λ* of the h-BN/MT. This phenomenon can be attributed to the rise in the content of h-BN; more thermal conductive pathways can be formed which leads to the elevation of the *λ* of h-BN/MT. As the h-BN loading increases from 0 wt.% to 10 wt.%, the thermal conductivity growth rates of the 50 nm and 10 μm doped mica tapes are essentially identical. This is primarily because the low doping levels in both cases provide new thermal conduction pathways, resulting in comparable thermal conductivity enhancements. When the h-BN loading increases to 15 wt.%, the thermal conductivity enhancement of the 50 nm filling is lower compared to the 10 μm filling in mica tape. This is primarily because the smaller 50 nm particle size allows some h-BN particles to enter the gaps between mica sheets. This prevents effective bridging to form a complete thermal conduction pathway. In contrast, the larger 10 μm particles can better bridge these gaps and are less likely to be covered by the mica sheets. When the h-BN doping content ranges from 10 wt.% to 15 wt.%, the slope of the enhanced *λ* in 50 nm doped mica tapes is significantly steeper than that in 10 μm tapes. This occurs because the quantity of 50 nm h-BN accumulates sufficiently to connect previously relatively isolated smaller boron nitride particles in series, forming a greater number of thermal conduction pathways. In contrast, the larger particle size of 10 μm h-BN results in more stable thermal conduction pathways, leading to a more consistent slope in the increase in *λ*. As the h-BN filler content increases from 15 wt.% to 20 wt.%, the improvement in *λ* of the 50 nm doped mica tape diminishes. This is primarily due to agglomeration effects occurring at higher concentrations when particle size is smaller, which impedes the formation of thermal conduction pathways. Simultaneously, the thermal conductivity enhancement of the 10 μm doped mica tape also shows a decrease. This occurs because the 10 μm h-BN particles had already established most of the thermal pathways at earlier concentrations, leaving limited capacity for additional pathways to form at higher concentrations. And when the filling amount of h-BN is from 10 wt.% to 15 wt.%, the increase in the content of h-BN makes it easier to lap, and the propagation path of phonons is transferred from the mica flake to the h-BN thermal conductivity network, which makes the thermal conductivity enhancement in this stage more obvious. When the filling amount is from 15 wt.% to 20 wt.%, the increment of the thermal conductivity enhancement rate is smaller in this stage because the h-BN size is smaller and it is easier to cause agglomeration when the concentration is larger. Secondly, as shown in Figure 4b, when the filling amount of h-BN (10 μm) ranges from 0 wt.% to 15 wt.%, the enhancement rate of the *λ* of the h-BN/MT is more stable, which is primarily attributable to the fact that, due to their larger dimensions, the h-BN grains exhibit a greater probability to overlap one another, and is less affected by the interfacial thermal resistance. When the filling amount is from 15 wt.% to 20 wt.%, the growth of *λ* of the composite material slows down, which is due to the larger size of h-BN, and the saturation of the material’s internal thermal pathway is attained at a content of 15 wt.%, thereby inducing a decline in the growth rate of the composite material’s *λ* as the h-BN content continues to increase. The thermal conductivity of BN-filled mica tapes with two particle sizes increased by 91.95% and 101.01%, respectively. Compared to the modified mica tape studied by Zhang [42] (which achieved a 75.53% increase in thermal conductivity), this material demonstrates a significant improvement. At low filler content, the thermal conductivity of the two mica tapes with different particle sizes doped in this study is comparable to that of the mica tape prepared by Feng [43], but the manufacturing process is simpler in comparison. Compared to the Al_2_O_3_-doped mica tape prepared by Zhou [44], at similar filler contents, the thermal conductivity of the 50 nm-sized doped mica tape prepared in this study is slightly lower than that of the mica tape reported in You’s work, while the 5 μm-sized doped mica tape exhibits higher thermal conductivity than the mica tape prepared by You. This further demonstrates the impact of different particle sizes on the thermal conductivity of doped mica tapes.

Figure 4c demonstrates the thermal conductivity mechanism diagram of the small-size h-BN/MT, and we can find that its state inside the EP is more dispersed at lower h-BN concentrations, which leads to the inability to constitute a complete thermal conductivity pathway, and thus the thermal conductivity enhancement is not obvious. When the amount of h-BN is sufficiently high, it can constitute a good thermal conductive pathway. Figure 4d demonstrates the thermal conductivity mechanism diagram of the large-size h-BN/MT, which shows that the large-size h-BN are more easily lapped with each other to form the thermal conductivity pathway. As illustrated by the schematic diagram, smaller h-BN grains are more likely to have isolated monomers at the same mass fraction. This h-BN is unable to effectively establish a thermal conductivity pathway. Conversely, larger h-BN grains exhibit a reduced tendency for isolated h-BN. This phenomenon also elucidates the higher value of the *λ* of large-size h-BN in comparison to that of small-size h-BN at equivalent concentrations. In addition, the size of the h-BN also affects its ability to get between the fiberglass cloth, allowing the thermal path to run through both the fiberglass cloth and the EP. This also means that h-BN is not as big as possible but needs to be considered compatible with the fiberglass cloth. Considering the above factors as well as the experimental results, micron-sized h-BN is more suitable to be used for reinforcing the *λ* of mica tape.

**Figure 5:**
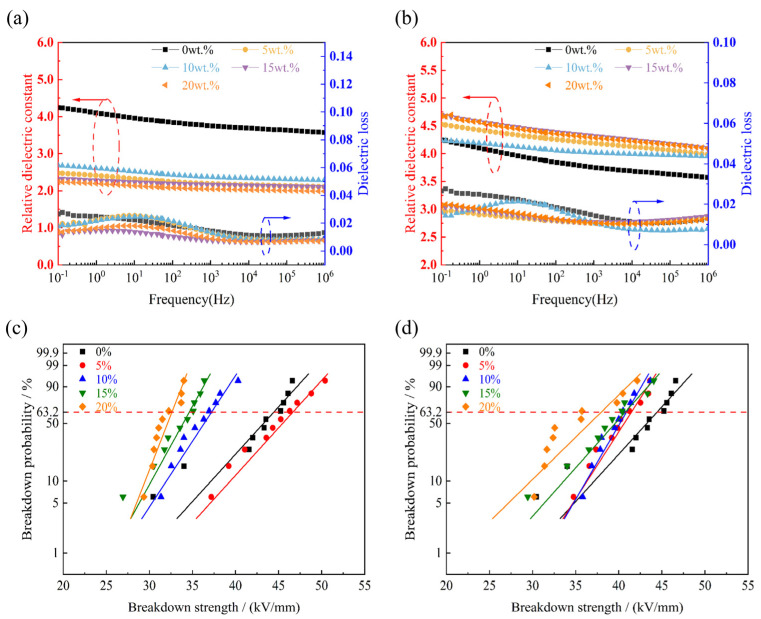
The *ε_r_* and tan*δ* of h-BN/MT with two sizes: (**a**) 50 nm, (**b**) 10 μm. The *E_b_* for different sizes of h-BN/MT (**c**) 50 nm, (**d**) 10 μm.

The *ε_r_* of the h-BN/MT gradually decreases with the increase in frequency Figure 5a,b. When the frequency is low, the electric field changes slowly, and the dipole within the h-BN/MT composite exhibits sufficient response time to electric field variations while maintaining synchronous polarization, so it is more sufficiently polarized and has a larger *ε_r_* at low frequencies (the *ε_r_* of mica tape filled with 50 nm particles is approximately 2.4, while that of 10 μm particles is around 4.5). In the mid-frequency range, phenomena such as dipole polarization relaxation occur, leading to a lag in the polarization response. Consequently, there has been a decline in the *ε_r_*. In the high-frequency region, the electric field changes extremely rapidly the electron-induced charge in the h-BN/MT lags behind the electric field’s rate of variation, resulting in a polarization that does not respond adequately to changes in the electric field, and hence a decrease in the *ε_r_* (the *ε_r_* of mica tape filled with 50 nm particles is approximately 2.1, while that of 10 μm particles is around 4). As shown in Figure 5a, The doped h-BN/MT with a size of 50 nm exhibits significantly lower *ε_r_* compared to undoped mica tape, which may be mainly on account of that nanoscale h-BN/MT can effectively fill the defects therein and reduce the space charge polarization effect at the interface, while the nanoscale h-BN is also likely to impede the migration of carriers, which reduces the *ε_r_* of the doped tape. The *ε_r_* of the h-BN/MT (10 μm) demonstrates an increase relative to unmodified mica tape, potentially attributable to larger h-BN grains easily establishing interconnected thermal conduction networks, which can be evidenced by the *λ*. At the same time the creation of the thermal conductive pathway by the h-BN is conducive to the migration of the polarized charge under the action of the electric field, which results in a rise in the *ε_r_* of the h-BN/MT (Figure 5b). In addition, with larger size, space charge is more likely to accumulate at the interface between h-BN and EP, forming an additional dipole moment, which results in a rise in the *ε_r_* of the h-BN/MT. Concurrently, the utilization of large-size h-BN may result in the introduction of an excessive number of defects at its edges. These defects can function as polarization centers, thereby enhancing the dipole response and increasing the *ε_r_*. The tan*δ* of all the samples is found to be similar, with a value lower than 0.02, which is in accordance with the requirements for practical engineering applications.

The *E_b_* of mica tape with different sizes and different h-BN fillings were analyzed using the Weibull distribution [45], and the calculation equations are shown in Equations (S3)–(S5), and Tables S1 and S2 show the parameters of the Weibull distribution. The *E_b_* of h-BN/MT (50 nm) exhibits a non-monotonic trend with increasing h-BN filler content, first rising then declining (Figure 5c). The increase in the *E_b_* of the h-BN/MT at 5 wt.% may be due to the fact that the defects within the dielectric can be better filled at lower h-BN loading levels, which coincides with the possibility of the decrease in the *ε_r_*, as stated above. However, when the filling amount of h-BN gradually rises, the constituted thermal conductive pathways will simultaneously provide for the development of electrical trees, and thus the *E_b_* of the h-BN/MT gradually decreases with increasing h-BN content. When the h-BN filler content is 20 wt.%, the mica tape achieves its lowest breakdown electric field strength of 32.48 kV/mm. The *E_b_* of the h-BN/MT with a size of 10 μm decreases with the gradual increase in the filling amount of h-BN, which is mainly due to the fact that the introduction of h-BN with a large-size produces many defects and constitutes a large number of intact thermal conduction paths, both of which provide convenient conditions for the formation of the electric tree, and thus the *E_b_* of the h-BN/MT decreases. In addition, regardless of the size, high mass fraction of h-BN tends to trigger agglomeration, which leads to uneven dispersion of h-BN in the mica tape and causes uneven accumulation of charge, thus reducing the *E_b_* (Figure 5d). In addition, the *E_b_* of all the samples prepared in this paper are higher than 32 kV/mm, which satisfies the demands of practical engineering applications (25 kV/mm).

**Figure 6:**
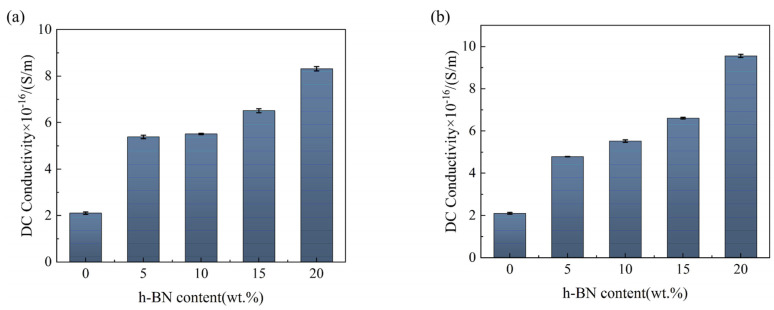
The *γ* of h-BN/MT of two sizes: (**a**) 50 nm, (**b**) 10 μm.

The *γ* of h-BN/MT of two sizes was tested at room temperature (25 °C) and 10 kV/mm voltage level (Figure 6). The *γ* of h-BN/MT of both sizes increases with the increase in h-BN content. The main reason is that the introduction of h-BN increases the defects in the mica tape, which leads to an increase in the number of carriers transported, resulting in an increase in the *γ* of the h-BN/MT. In addition, as the proportion of h-BN within the composite material increases, the number of thermal conductive pathways within the mica tape also increases, which also makes the movement of carriers easier, leading to an increase in *γ*. However, as a whole the *γ* of all the samples is below 1 × 10^−15^ S/m, far below the national standard requirement 1 × 10^−12^ S/m. While enhancing thermal conductivity, it also ensures that dielectric properties meet practical requirements. It is noteworthy that the relative dielectric constant of mica tape modified with 50 nm-sized BN particles has decreased, which may offer better performance in certain specialized application environments.

### 3.3. Modification of h-BN/MT Thermal Conductivity Models for Different Sizes

At present, the thermal conductivity model for h-BN/MT does not take into account the effect of h-BN size selection on the *λ* of mica tape. In addition, mica tape is composed of three parts of materials, so the conventional first-order thermal conductivity model cannot be used to accurately predict the *λ* of mica tape. Therefore, a second-order thermal conductivity model of h-BN/MT with different sizes is constructed in this paper with h-BN size as the core parameter, which is of impact for the prediction of *λ* in mica tape.

Before modifying the model, the following prerequisites were established: First, h-BN is uniformly dispersed in the EP. Second, the mica paper is structurally compact with no internal gaps. Third, the EP impregnates the fiberglass cloth as well as the mica paper well. The h-BN-modified EP is treated as a whole in this model, and together with the mica paper and fiberglass cloth, it forms the three parts of the mica tape. Common thermal conductivity models include, Maxwell–Eucke model, Lewis–Nielsen model, Halpin–Tsai model, and Percolation Theory model [46,47,48,49,50], etc., among which the Halpin–Tsai model focuses on the consideration of the morphological characteristics of the thermally conductive filler, and therefore, in the h-BN-modified epoxy resin system Halpin–Tsai model was adopted as shown in Equations (1)–(3).

(1) $$K_{f}=K_{m}\left(\frac{1+\zeta \eta V_{p}}{1-\eta V_{p}}\right)$$

where *K_f_* is the *λ* of the composite, *K_m_* is the *λ* of the matrix, *ζ* is the shape factor, *η* is the thermal conductivity comparison factor between the filler and the matrix, and *V_p_* is the volume fraction of the filler.

(2) $$\;\eta =\frac{K_{p}/K_{m}-1}{K_{p}/K_{m}+\zeta }$$

where *K_p_* is the *λ* of the filler, *K_m_* is the *λ* of the matrix, and *ζ* is the shape factor.

(3) $$\;\zeta =2\left(L/D\right)$$

where *L*/*D* is the filler aspect ratio.

Subsequently, the three components of modified h-BN-modified epoxy resin, mica paper as well as fiberglass cloth were calculated again using the Series model as shown in Equation (4).

(4) $$\frac{1}{K_{f}}=\sum \frac{V_{i}}{K_{i}}$$

where *K_f_* is the *λ* of the composite, *V_i_* is the volume fraction of the whole occupied by h-BN/EP, mica paper, and fiberglass cloth, respectively, and *K_i_* is the *λ* of the above three components.

**Figure 7:**
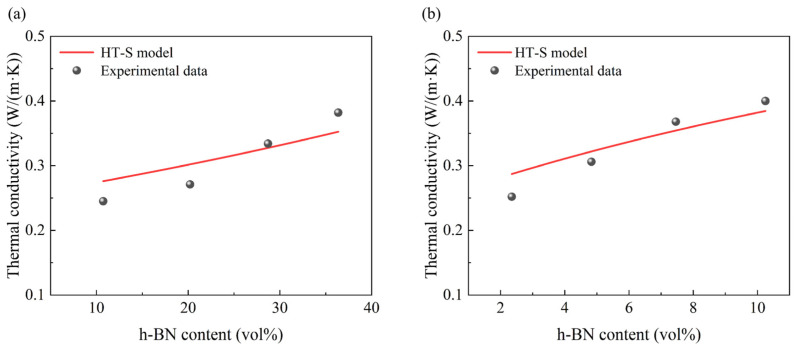
Comparison of HT-S model thermal conductivity with experimentally obtained thermal conductivity: (**a**) 50 nm, (**b**) 10 μm.

The above model is named as HT-S model and the model calculation process is shown in Equations (S1) and (S2). The *λ* calculated from this model was then compared with the experimentally derived data as shown in Figure 7. The difference between the *λ* calculated by the HT-S model and the experimentally obtained *λ* is not significant. Despite the composition of mica tape consisting of three materials, the HT-S model has been demonstrated to predict the *λ* of mica tape with greater accuracy. Base on HT-S model, it can be observed that the size of h-BN has a significant effect on the *λ* of mica tape, and the selection of different sizes of h-BN will produce different effects on the *λ* of mica tape.

## 4. Conclusions

In this paper, h-BN/MT with different sizes (50 nm, 10 μm) were prepared by physical co-mingling and hot pressing techniques, and it was found by microscopic characterization that h-BN formed a complete thermally conductive pathway in EP, which provided a good pathway for the circulation of heat. The thermal conductivity test shows that under the condition of 10 μm h-BN size and 20% by weight filling, the *λ* of the mica tape reaches 0.4 W/(m·K), representing a 101.01% increase compared with the unmodified mica tape. In the study of dielectric properties, the *ε_r_* of the h-BN/MT decreases gradually as the frequency increases. However, the tan*δ* of all samples is less than 0.02, indicating excellent dielectric properties. The breakdown field strength test results show that the *E_b_* of h-BN/MT of two sizes show a decreasing trend with the increase in h-BN content, but all of them are kept above 32 kV/mm, which can be applied in practical engineering. The *γ* of all samples was below 1 × 10^−15^ S/m with good insulating properties. The advantages and disadvantages of h-BN/MT with different sizes were found by studying the *λ*, dielectric, and *E_b_* of different h-BN/MT: h-BN/MT with a size of 50 nm have a smaller enhancement of *λ* but good dielectric and *E_b_*, while the other h-BN/MT have a more pronounced enhancement of *λ* but a decrease in dielectric and *E_b_*. In addition, a second-order thermal conductivity model was developed, which can calculate the *λ* of h-BN/MT doped with different sizes of h-BN. This study can provide some guidance for the selection of h-BN size during the preparation of mica tape composites.

## Data Availability

The original contributions presented in this study are included in the article/Supplementary Materials. Further inquiries can be directed to the corresponding authors.

## Supplementary Materials

The following supporting information can be downloaded at: https://www.mdpi.com/article/10.3390/ma19091821/s1, Figure S1: Comparison of predicted thermal conductivity values with actual values; Table S1: the values of two parameters of h-BN/mica tape with 50 nm particle size; Table S2: the values of two parameters of h-BN/mica tape with 10 μm particle size; Equation (S1): Halpin–Tsai model. Equation (S2): Series Model. Equation (S3): The median cumulative failure probability rank value. Equation (S4): The two-parameter Weibull distribution. Equation (S5): Shifting terms and natural logarithm processing. Reference [42] is cited in Supplementary Materials.
