# Supplementary material for: Effect of Micro and Nano Boron Nitride on Thermal Conductivity and Electrical Properties of Mica Tape
Source: Materials (Basel). 2026 Apr 29;19(9):1821. doi: 10.3390/ma19091821 (PMC13165072; doi:10.3390/ma19091821)
Supplement: Supplementary file 1 [file materials-19-01821-s001.zip › materials-4173079-supplementary.pdf]

Effect of Micro and Nano Boron Nitride on Thermal Conductivity and Electrical Properties of Mica Tape

Yu Feng [1,2], Minhao Tian [1,2], Xuesong Chen [1,2], Wenchao Zhang [1,2], Sergey A. Maksimenko [3], Dong Yue [1,2,*] and Yuanhang Yao [1,2,*]

[1] Key Laboratory of Engineering Dielectrics and Its Application, Ministry of Education, Harbin University of Science and Technology, Harbin 150080, China
[2] School of Electrical and Electronic Engineering, Harbin University of Science and Technology, Harbin 150080, China
[3] Institute for Nuclear Problems, Belarusian State University, 220006 Minsk, Belarus
* Correspondence: yuedong@hrbust.edu.cn (D.Y.); yhyao@hrbust.edu.cn (Y.Y.)

S1. HT-S model calculation process

The model is divided into two parts: firstly, the h-BN-doped epoxy resin system is used in the Halpin–Tsai model, and then the h-BN-doped epoxy resin system, mica paper and glass fiber cloth are used in Series model for calculating the final thermal conductivity of the mica tape. The specific calculation process is as follows:

(1) Halpin–Tsai model

$$K_f = K_m\left(\frac{1+\zeta\eta V_p}{1-\eta V_p}\right)$$

$$\eta = \frac{K_p/K_m - 1}{K_p/K_m + \zeta} \tag{S1}$$

$$\zeta = 2\left(L/D\right)$$

Where K_f is the thermal conductivity of the composite, K_p is the thermal conductivity of the filler, K_m is the thermal conductivity of the matrix, ζ is the shape factor, η is the thermal conductivity comparison factor between the filler and the matrix, and V_p is the volume fraction of the filler, and L/D is the filler aspect ratio.

(a) Calculate the h-BN/EP at 50 nm

The ζ for 50 nm was calculated to be 0.5, K_p is the h-BN thermal conductivity of 180 W/(m·K), and K_m is the epoxy thermal conductivity of 0.2 W/(m·K). η was calculated to be 899/900.5.

(b) The process of calculating 10 μm h-BN/EP

The ζ for 10 μm was calculated to be 10, K_p is the h-BN thermal conductivity of 180 W/(m·K), and K_m is the epoxy thermal conductivity of 0.2 W/(m·K). η was calculated to be 899/910.

(2) Series Model

$$\frac{1}{K_f} = \sum \frac{V_i}{K_i} \tag{S2}$$

where K_f is the thermal conductivity of the composite, V_i is the volume fraction of the whole occupied by h-BN/epoxy resin, mica paper, and glass fiber cloth, respectively, and K_i is the thermal conductivity of h-BN/epoxy resin, mica paper, and glass fiber cloth.

Bringing in the thermal conductivity of h-BN-doped epoxy resin obtained above, the thermal conductivity of mica paper was 0.3 W/(m·K) and that of glass fiber cloth was 0.9 W/(m·K). h-BN-doped epoxy resin accounted for 51% of the volume fraction, mica paper accounted for 41.3% of the volume fraction, and the glass fiber cloth accounted for 7.7% of the volume fraction.

(3) Thermal conductivity model application

Using this model, we predicted the thermal conductivity of the BN-doped modified mica tape with a particle size of 1-3 μm prepared by Zhang [1] and compared the results with their experimental data, as shown in Figure S1.

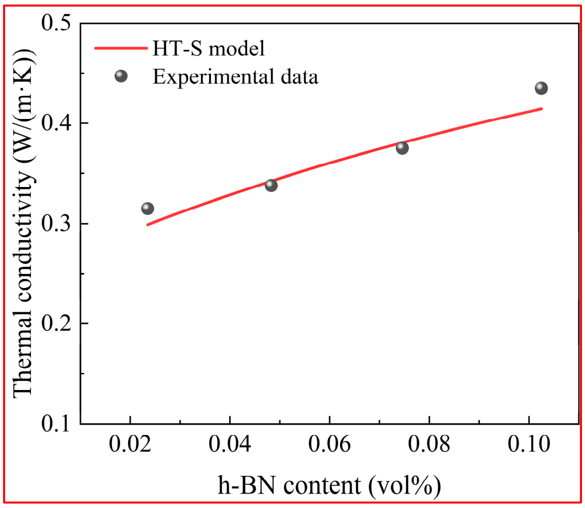

Figure S1. Comparison of predicted thermal conductivity values with actual values

Since this model is based on the Halpin–Tsai model, two issues arise: 1. In thermal conductivity predictions, it assumes all thermal conductive fillers are perfectly aligned in a single orientation, which is difficult to achieve in actual preparation processes. 2. It neglects interfacial effects. These two issues are precisely what cause the discrepancy between experimental and predicted values.

S2. Breakthrough Field Strength Test

The cumulative probability of failure calculation and two-parameter Weibull distribution analysis are performed for the breakdown strength. The median cumulative failure probability rank value can be approximated by Eq. (S3):

$$P = \frac{i-0.5}{n+0.25} \tag{S3}$$

where i is the number of failure sequence; n is the sample capacity of the specimen.

The equation for the relationship between the breakdown strength and the cumulative breakdown probability used for the two-parameter Weibull distribution is shown in Equation (S4):

$$P(E) = 1 - \exp\left[-\left(\frac{E}{E_b}\right)^{\beta}\right] \tag{S4}$$

where $P(E)$ is the cumulative breakdown probability; E is the test breakdown strength; β is a parameter representing the dispersion of the breakdown strength value, the shape parameter; E_b is the characteristic breakdown strength of the specimen at P=63.2%. Shifting terms and natural logarithm processing for the equation of Eq. (S4) can obtain the equation:

$$\ln\left(-\ln\left(1-P\left(E\right)\right)\right) = \beta \ln E - \beta \ln E_b \tag{S5}$$

Based on the samples obtained from the breakdown tests, a linear fit of ln$E$ to ln(-ln(1-P(E))) using Eq. (S5) allows the intercept and slope to be obtained, which in turn allows the derivation of E_b and β values.

S3. The values of two parameters of h-BN/mica tape with 50 nm particle size

Table S1. The values of two parameters of h-BN/mica tape with 50 nm particle size

h-BN mass fraction	E_b	β

0 wt.%	43.85	12.48
5 wt.%	46.11	13.21
10 wt.%	36.86	14.74
15 wt.%	34.36	16.49
20 wt.%	32.48	22.40

S4. The values of two parameters of h-BN/mica strips with 10 μm particle size

Table S2. The values of two parameters of h-BN/mica tape with 10 μm particle size

h-BN mass fraction	E_b	β
0 wt.%	43.85	12.48
5 wt.%	41.16	16.94
10 wt.%	40.63	18.55
15 wt.%	40.13	11.62
20 wt.%	37.06	9.16

References

[1] Zhang Z, Feng Y, Wang D, et al. Thermal conductive network construction and enhanced thermal conductivity in mica tape composites for large generator insulation[J]. Composites Science and Technology, 2024, 254: 110671.
